# Non-optic glioma in adults and children with neurofibromatosis 1

**DOI:** 10.1186/s13023-017-0588-2

**Published:** 2017-02-15

**Authors:** Laura Sellmer, Said Farschtschi, Marco Marangoni, Manraj K. S. Heran, Patricia Birch, Ralph Wenzel, Jan M. Friedman, Victor-Felix Mautner

**Affiliations:** 1 Department of Medical Genetics, University of British Columbia, Vancouver, Canada; 2 Department of Neurology, University Hospital Hamburg-Eppendorf, Hamburg, Germany; 3 Diagnostic and Therapeutic Neuroradiology, University of British Columbia, Vancouver, Canada; 4 Department of Radiology, MRI Institute Altona, Hamburg, Germany

**Keywords:** Neurofibromatosis 1, Cohort study, Glioma, Adults, Children, Prospective

## Abstract

**Background:**

Non-optic gliomas occur in 5% of children with NF1, but little is known about these tumours in adults. We aimed to investigate progression, spontaneous regression and the natural history of non-optic gliomas in adults and compare these findings to the results found in children.

**Results:**

One thousand seven hundred twenty-two brain MRI scans of 562 unselected individuals with NF1 were collected at the NF outpatient department of the University Hospital Hamburg-Eppendorf between 2003 and 2015. The number of scans per patient ranged from one to 12; patients were followed for a median of 3.7 years. We identified 24 patients (4.3%) with non-optic gliomas. Median age at first scan with glioma was 21.2 years, much higher than in previous publications. Only seven of the 24 non-optic glioma patients were symptomatic. Five of 24 patients had multiple non-optic gliomas. Four individuals developed a new tumour, and 4 cases showed progression. The risk of new tumour development was 0.19% (95% confidence interval 0.06% to 0.52%) per patient year of follow-up for patients over 10 years. The rate of progressing non-optic gliomas per patient year of follow-up in the first 5 years after tumour diagnosis was 4.7% (95% confidence interval 1.5% to 12%).

**Conclusions:**

Non-optic gliomas are twice as common in an unselected cohort of NF1 patients as previously reported. This is likely due to increased frequency of diagnosis of asymptomatic tumours when routine MRIs are performed and a higher prevalence in older individuals.

## Background

Neurofibromatosis 1 (NF1) is an autosomal dominant disorder with an estimated incidence of 1 in 3000 live births [1]. It is caused by mutations in the *NF1* gene, a suppressor of the RAS kinase pathway. As a result of the NF1 protein’s ubiquitous expression and important role in cellular regulation, patients with mutations in the *NF1* gene develop e.g., neurofibromas and other neoplasms and pigmentary abnormalities of the skin [2].

Gliomas outside of the optic pathways are one of the most common causes of death in NF1 patients [3, 4]. Non-optic gliomas in NF1 patients are usually located in the brainstem or cerebellum and are almost exclusively reported in children, although non-optic gliomas are known to occur in adults with NF1 as well [5]. Approximately 5% of children with NF1 develop a non-optic glioma, a prevalence more than 100 times greater than in the general population [6]. Non-optic gliomas in NF1 patients are usually low-grade astrocytomas and often have a more benign course than in people without NF1 [7–9]. Their natural history is only incompletely understood [10]. The occurrence of non-optic gliomas has been found to be associated with the presence of optic gliomas in children with NF1 in some studies [11, 12].

MRI is commonly used to evaluate intracranial tumours in clinical as well as research settings [13]. MRI provides information on the presence, location, and size of the tumour and is a crucial tool in routine clinical care of gliomas in individuals with or without NF1.

In this study, we investigated the natural history, progression, and regression of non-optic gliomas in an unselected cohort of adults and children with NF1.

## Methods

### Patients

Between 2003 and 2015, all patients with NF1 diagnosed according to standard clinical criteria seen in the NF outpatient department of the University Hospital Hamburg–Eppendorf were offered brain MRIs [14]. Because patients were not selected for imaging on the basis of clinical symptoms, they are representative of the NF1 population seen in the clinic.

The ethical committees of the Medical Chamber in Hamburg and the Research Ethics Board of the University of British Columbia approved the study. Written consent was obtained from all study participants before study begin. All data were de-identified before analysis.

### MRI imaging

Lesions that were hyperintense in T_2_ without mass effect and enhancement were identified as unidentified bright objects (UBO). In order to distinguish gliomas from UBOs, we used the following criteria: location and size of a lesion, the presence of mass effect, the lesion being hyperintense in T_2_ and hypointense in T_1_, and evolution over time.

Based on the 1722 German clinical MRI reports, a list of patients was generated who had been diagnosed with non-optic gliomas. All brain MRIs from these patients were re-evaluated by two neuroradiologists in Canada (M.M. and M.K.S.H.), and the presence of non-optic gliomas in each individual patient was established by consensus using the criteria described above.

In NF1 patients without anomalies on head MRI, follow-up imaging was performed in 2-year intervals. In patients with asymptomatic non-optic gliomas, imaging was repeated after 1 year. In patients with clinically or radiologically progressive non-optic gliomas, re-imaging was performed at 6-month intervals. Treatment decisions were based on location and severity of symptoms in symptomatic patients, and based on location and rate of tumour growth in asymptomatic patients. Surgical resection was the treatment of choice if it could be done safely; if the tumour was unresectable, chemotherapy was offered.

Tumour volume was calculated by using a box model. New appearance of a glioma was defined when an individual had a tumour on at least one MRI in an area that was well imaged but no tumour was seen in at least one previous scan. Progression of a non-optic glioma was defined as an increase in volume of at least 30% per year.

### Descriptive statistical analysis

Patients were divided into 10-year age groups and only counted once per age group. Patients who were scanned in more than one decade of life were counted once in each age group in which a scan was performed. Patients with more than one glioma present were counted only once per age group. Ninety-five percent confidence intervals of the percentages were calculated as ± 1.96 standard deviations of a Poisson distribution.

Tumour volume in younger patients was compared to that in older patients with a Mann–Whitney *U* test.

Ninety-five percent confidence intervals for rates of newly appearing tumours and progressing tumours were calculated using Wilson’s method with continuity correction.

Results were considered to be significant if *p* ≤ 0.05.

## Results

### Demographics

**Fig. 1:**
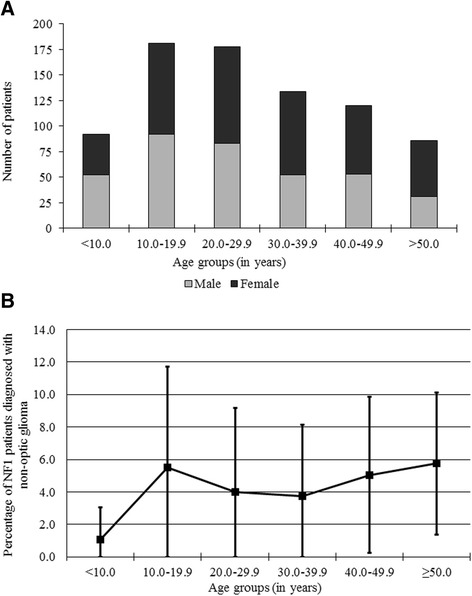
Demographics and percentage of NF1 patients affected by non-optic glioma per age group. **a** Number of male and female NF1 patients per age group. Each person is counted once per age group, regardless of number of scans in that age group. Individuals may appear in more than one age group if scanned in more than one age range. **b** The overall prevalence of non-optic glioma appears stable in adulthood. Each person is counted once per age group, regardless of number of scans in that age group. Individuals may appear in more than one age group if scanned in more than one age range. Error bars are 1.96 standard deviations of a Poisson distribution

**Fig. 2:**
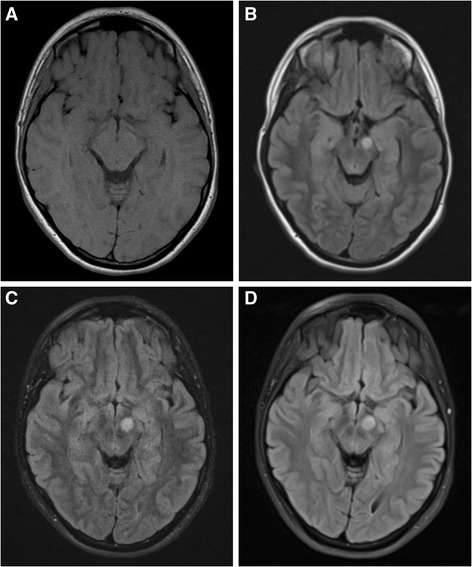
Newly-appearing glioma in the left cerebral peduncle in Patient 19. **a** There is no visible glioma on the patient’s first scan. **b** 4 years later, an enhancing glioma has appeared in the left cerebral peduncle measuring 0.5 cm^3^. **c** 5 years after the initial glioma-free scan, the glioma has increased to a volume of 0.8 cm^3^. **d** 7 years after the initial scan, the glioma measured 1.3 cm^3^. All images shown are FLAIR sequences. The patient remained asymptomatic during follow-up and also has an optic glioma (not visible in these images)

A total of 562 patients with NF1, 264 males and 298 females (1.0:1.1 ratio), were included in this study (see Figs. 1a and 2). One thousand seven hundred twenty-two MRI scans were performed, with a median of two scans per person (range, 1 to 12 scans) and a median follow-up time of 3.7 years (range, 0 to 13.0 years). At the time of the MRI scan, their ages ranged from 0.4 to 72.8 years.

During the study period, 51 patients were lost to follow-up and 24 died of reasons unrelated to non-optic gliomas, equaling a dropout rate of 13.3%.

### Number of patients with non-optic glioma by age group

A diagnosis of non-optic glioma was made on clinical reading of the brain MRI images in 27 NF1 patients. In 24 of these cases, the study neuroradiologists (M.M. and M.K.S.H.) were able to confirm the definite presence of a non-optic glioma using the study criteria.

Thus, a total of 24 (4.3%) of the 562 NF1 patients were diagnosed with non-optic glioma. The prevalence of individuals diagnosed with non-optic glioma per 10-year age group is shown in Fig. 1b. Follow-up of the 24 non-optic glioma patients ranged between 0 years and 11.8 years, with a median of 5.0 years of follow-up and a combined total of 134 years of follow-up.

### Clinical description of gliomas

An overview of all non-optic glioma cases is provided in Table 1. There were a total of 32 gliomas in 24 patients. Nineteen patients had one glioma, two patients had two non-optic gliomas, and three patients had three non-optic gliomas. Only seven of the 24 non-optic tumour patients were symptomatic. Individual tumour volumes ranged from 0.04 cm^3^ to 98.4 cm^3^, with a median volume of 1.6 cm^3^.

**Table 1:** Clinical features of non-optic gliomas in NF1 patients

Patient number	Sex	Age at first scan with glioma (in years)	Symptoms	Histology	Location	Status	Treatment
1	M	20.9	Headache	PA Grade 1	Frontal	Decreased size	Surgery
2	F	41.6	No		Temporal	Decreased size	Chemotherapy (breast cancer)
3	F	48.6	No		Brainstem	Stable	
4	F	50.8	No		Temporal	No follow up	
5	F	23.4	Seizures		Brainstem	Stable	
6	F	10.0	Seizures, headache		Cerebellum	Stable	
7	M	31.3	No		Brainstem	Stable	
8	M	13.1	No		Frontal	Stable	
9a^b^	M	44.6	Ataxia		Cerebellum	Stable	
9b^b^	M	44.6	Ataxia		Cerebellum	Stable	
9c^ab^	M	50.5	Ataxia	PA Grade 1	Cerebellum	Increased, then decreased size	Surgery
10	M	6.5	No	PA Grade 1	Brainstem	Increased size & enhancement	Chemotherapy, biopsy
11^ab^	F	34.9	No		Brainstem	No follow up	
12a	F	16.4	No	PA Grade 1	Corpus Callosum	Increased enhancement	Incomplete resection before study begin
12b^a^	F	25.3	No		Brainstem	Stable	
13	F	38.5	No		Cerebellum	Stable	
14	M	17.2	Seizures, headache	PA Grade 1	Cerebellum	Stable	Surgery
15	M	21.3	No	PA Grade 1	Corpus Callosum	Decreased size & enhancement	Surgery
16	M	42.5	No		Temporal	Stable	
17	M	42.7	Seizures	DNET	Cerebellum	Stable	Incomplete resection before study begin
18a	F	14.8	No	PA Grade 1	Corpus Callosum	No follow up	Surgery
18b	F	14.8	No		Thalamus	No follow up	
19^ab^	F	18.3	No		Brainstem	Increased in size	
20	F	16.0	No		Internal capsule	Decreased enhancement	
21	F	39.8	No		Cerebellum	Stable	
22a	F	10.9	No		Cerebellum	Decreased enhancement	
22b	F	10.9	No		Cerebellum	Increased, then decreased in size	
22c	F	10.9	No		Cerebellum	Stable	
23	M	17.4	No		Brainstem	Increased size & enhancement	
24a	M	18.1	Double vision, nystagmus	PA Grade 1	Brainstem	Decreased size	Surgery
24b	M	18.1	Double vision, nystagmus		Corpus Callosum	Increased size	
24c	M	18.1	Double vision, nystagmus		Cerebellum	Stable	

*Abbreviations*: *PA* pilocytic astrocytoma, *M* male, *F* female, *DNET* dysembryoplastic neuroepithelial tumour

^a^denotes patients with newly-appearing tumours

^b^denotes patients with concurrent optic gliomas

Twenty-five of 32 tumours enhanced after administration of gadolinium. Eight tumours showed avid enhancement, four tumours showed diffuse enhancement, and enhancement was patchy or peripheral in 13 tumours. Enhancement increased in three tumours and decreased in another three tumours. These changes were not associated with alterations in clinical status or other radiological features.

Nine patients received treatment for their gliomas (Table 1): eight patients underwent surgery and one patient received vincristine and carboplatin following a biopsy. None of these nine patients had recurrence or further growth of their non-optic gliomas after treatment.

Histology was available on nine tumours. Eight tumours were pilocytic astrocytomas (PAs) (WHO Grade 1) and one was a dysembryoplastic neuroepithelial tumour (DNET). DNETs are of glioneuronal origin and are classified as WHO Grade 1. There were no higher-grade tumours.

Three patients with non-optic gliomas had concurrent optic gliomas (Patients 9, 11 and 19 in Table 1). There was no significant association between the presence of non-optic gliomas and optic gliomas (*χ* ^2^ = 0.31, *p* = 0.57).

### Newly diagnosed non-optic gliomas

There were four individuals with newly-appearing gliomas (see Fig. 2) (Patients 9c, 11, 12b, and 19 in Table 1). Since glioma prevalence is stable in patients over 10 years, we determined the rate of new tumours appearing during 2111 years of follow-up of patients age 10 and older. Four new tumours appeared, resulting in a rate of 0.19% (95% confidence interval 0.06% to 0.52%) of new non-optic glioma development per patient year of follow-up. There were no newly-appearing non-optic gliomas in the 266 years of follow-up of children younger than 10 years.

### Progressing gliomas

**Fig. 3:**
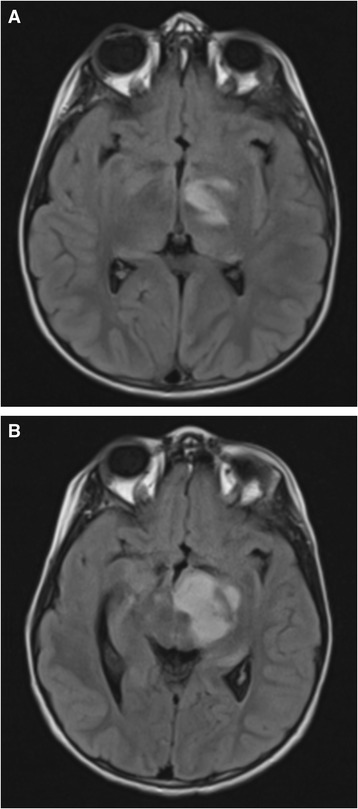
Progressing glioma in Patient 9. **a** First scan with glioma present in the left cerebral peduncle and left thalamus on FLAIR sequence. **b** Another scan performed 2 years after the previous one (again FLAIR sequence). The glioma has drastically increased in size and was treated with chemotherapy 1 month after this image was taken

We identified glioma progression in four individuals (Fig. 3) (Patients 10, 19, 23, and 24b in Table 1). The rate of progressing non-optic gliomas per patient year of follow-up in the first 5 years after tumour diagnosis was 4.7% (95% confidence interval 1.5% to 12%). No progression events were observed in the 48.1 patient years of follow-up that were more than 5 years after diagnosis. Tumours in four of 21 non-optic glioma patients progressed (three patients did not have follow-up after tumour diagnosis).

### Regressing gliomas

There were no spontaneously regressing gliomas in our study.

### Correlation of presence non-optic gliomas with presence of UBOs

A study by Griffiths et al. suggested that gliomas might arise from unidentified bright objects (UBOs) in children with NF1 [15]. We focused on the 4 patients with newly-appearing tumours (Patients 9, 11, 12, and 19 in Table 1) and assessed if they had UBOs in the location where the glioma later appeared.

None of the four non-optic glioma patients with newly-appearing gliomas had UBOs at the location of their tumour before it appeared.

## Discussion

This study comprises the world’s largest collection of unselected brain MRIs of NF1 patients reported to date. Twenty-four (4.3%) of 562 patients were diagnosed with non-optic gliomas. This overall prevalence is 1.5× to 3× higher than in previously published prospective studies (Table 2) [11, 16–21].

**Table 2:** Published prospective and retrospective studies assessing the prevalence of non-optic gliomas in NF1

Authors	Study type	Cohort size	Age range of cohorts (in years)	Median age at diagnosis	Number of tumour patients	Rates of tumours	How were tumour patients identified?
Cnossen et al. [16]	P	150	0–18	Unknown	4	2.70%	Symptomatic patients
Zöller et al. [17]	P	70	20–81	Unknown	1	1.43%	Cross-referencing with cancer registry
Friedman and Birch [11]	R	684	0–73	<6 years	25	3.65%	MRI/CT*
McGaughran et al. [18]	R	523	0–74	Unknown	12	2.29%	Symptomatic patients
Menor et al. [19]	R	72	0.8–14	Unknown	8	11%	MRI*
Seminog and Goldacre [20]	R	6739	0–80+	Unknown	322	4.78%	Hospital records
Varan et al. [21]	R	473	0–33	8 years	11	2.32%	Symptomatic patients

*denotes studies for which convenience MRI/CT images were used

This difference can be explained in part by the fact that the majority of non-optic glioma patients are asymptomatic (seven of our 24 glioma patients were symptomatic) and could only be detected by offering MRIs to all NF1 patients, as was done in our study.

The median age at first scan in this study is 21.2 years -- much higher than the usual age at diagnosis of non-optic gliomas in NF1 patients (Table 2).

Since our study uses age at first scan with glioma and not age at glioma diagnosis to infer prevalence, cases in younger age groups might be underrepresented. Additionally, the majority of adult NF1 patients with gliomas are asymptomatic and some symptoms of brain tumours are also common in NF1 patients without brain tumours (e.g., headache). Even though it is known that NF1 patients have a lifelong elevated risk for tumour development, brain MRIs are not currently recommended for tumour surveillance in NF1 patients of any age as part of regular clinical care [7, 22].

The largest group of adult NF1 patients with non-optic gliomas was described by Gutmann et al. [5] Only three of 15 tumours for which histology was available were Grade 1, compared to nine of nine in our study. This may be explained by Gutmann et al. only reporting symptomatic tumours, which are more likely to be high-grade than asymptomatic tumours. Other authors reported three adult cases, two of which were asymptomatic [23].

Intracranial gliomas in NF1 patients are usually associated with a better outcome than those diagnosed in the non-NF1 setting [24, 25]. Most of the tumours biopsied in this study were PAs -- the most common histological type of intracranial tumour [26]. The 5-year survival rate of NF1 patients with PA was estimated to be 85% [25]. However, this was calculated for patients who underwent surgery for their gliomas, so the true 5-year survival rate is likely much higher than 85%. In further support of this, none of our brain tumour patients with or without biopsy-proven PA died during a cumulative 134 patient years of follow-up.

While malignant transformation of low-grade non-optic gliomas has been described in NF1 patients [27, 28], this appears to be infrequent [29]. We did not see any cases of malignant transformation in this study.

The majority of non-optic gliomas in children with NF1 reported in the literature have been located in the posterior fossa [9, 30]. We observed the same thing in our study. We also found that the majority of non-optic gliomas in adults with NF1 were located in the posterior fossa. Previous reports suggested that these tumours usually occur in other locations in adults [5], but previous studies of non-optic gliomas in adults with NF1 have been small and focused on symptomatic patients. Most of the patients in our study were asymptomatic.

There were five patients with multiple non-optic gliomas. These lesions could either be multiple primary tumours, or multifocal gliomas. Gliomas in NF1 patients were shown to be multifocal [25]; however, it is impossible to distinguish between multifocal and multiple primary tumours without performing molecular analyses. The chance of having 1 non-optic glioma according to our results is 4.3%; therefore we expected to find one individual with two primary non-optic gliomas and 0 individuals with three primary non-optic gliomas by chance in a series of this size. We found two patients with two gliomas and three patients with three gliomas each. Even though these numbers are small, they support the possibility that a patient is more likely to develop additional non-optic tumours if he or she has already developed one. There is a correlation between the occurrence of optic and non-optic gliomas in people with NF1 [11]; however, a correlation between the development of non-optic gliomas has not previously been suggested. There are case reports of NF1 patients with more than one non-optic glioma, and all of these patients were symptomatic [31]. Only two of the five patients in our study showed symptoms, so NF1 patients can have multiple brain tumours and remain asymptomatic. A publication assessing prognostic factors for NF1 patients with brain tumours found that having multiple tumours is not a risk factor for death [32]; however, it did not distinguish between optic and non-optic tumours.

Tumours in adults with NF1 are said to be higher-grade and carry a worse prognosis than those in children with NF1 [32, 33]; however, we did not find any higher-grade tumours in adults. This difference probably reflects the fact that adults were only included in previous studies if they had aggressive tumours, whereas our study included individuals independent of the presence of symptoms.

In order to compare tumour volumes of younger and older non-optic glioma patients, we separated the cohort into two groups, using the median age at diagnosis (21.2 years) as cut-off. Tumours in patients diagnosed before 21.2 years had a significantly greater volume than those in patients diagnosed later (Mann–Whitney *U* test, *p* = 0.02). This is counterintuitive if most tumours in adults arose in childhood or adolescence and tumours only grow in young patients. However, a similar observation has been made for plexiform neurofibromas in people with NF1 -- the most rapid growth occurs during childhood but the tumour volume is inversely correlated with age [34]. In addition to having larger tumours, seven of the nine individuals requiring treatment of their non-optic tumour were diagnosed before 21.2 years of age. One possible explanation for the differences in tumour volume between children and adults is that large tumours in childhood require treatment or have fatal consequences, thus only leaving patients with small indolent tumours to be identified later.

One of the limitations of our study is that patients referred to the NF clinic are probably not representative of the NF1 patient population as a whole. Patients with milder manifestations may be less likely to be referred than those more severely affected. In addition, patients with e.g., brain tumours are more likely to get follow-up scans than tumour-free patients. Another limitation is that even though all parents were offered brain MRIs for their children, parents of asymptomatic children might be more likely to refuse MRI, which requires sedation, than parents of symptomatic children. Therefore, this study is probably biased towards patients with a more severe phenotype. It is important to note that there were patients seen in the NF outpatient department who had higher-grade tumours but were not included in this study.

There is currently no recommended head MRI screening protocol for patients with NF1, whether or not they have non-optic gliomas. We screen NF1 patients without non-optic gliomas every 2 years, patients with asymptomatic gliomas every 12 months, and patients with symptomatic gliomas every 6 months. A much larger longitudinal cohort study would be necessary to determine if this is the optimal approach.

Non-optic gliomas may occur in children as well as adults with NF1, and these tumours should be considered as a possible cause of neurological symptoms that develop in NF1 patients of any age.

## Conclusion

We determined the rate of appearance, progression and spontaneous regression in an unbiased NF1 cohort. This is the largest prospective study of unselected brain MRIs ever published and also the first study to compare glioma frequency and natural history in an unselected cohort with an age range this wide. Our data should be considered when counselling adults with NF1 who have a glioma.
